# Obstructions to Sampling Qualitative Properties

**DOI:** 10.1371/journal.pone.0135636

**Published:** 2015-08-19

**Authors:** Arne C. Reimers

**Affiliations:** Life Sciences Group, Centrum Wiskunde & Informatica, Amsterdam, Netherlands; Semmelweis University, HUNGARY

## Abstract

**Background:**

Sampling methods have proven to be a very efficient and intuitive method to understand properties of complicated spaces that cannot easily be computed using deterministic methods. Therefore, sampling methods became a popular tool in the applied sciences.

**Results:**

Here, we show that sampling methods are not an appropriate tool to analyze qualitative properties of complicated spaces unless **RP** = **NP**. We illustrate these results on the example of the thermodynamically feasible flux space of genome-scale metabolic networks and show that with artificial centering hit and run (ACHR) not all reactions that can have variable flux rates are sampled with variables flux rates. In particular a uniform sample of the flux space would not sample the flux variabilities completely.

**Conclusion:**

We conclude that unless theoretical convergence results exist, qualitative results obtained from sampling methods should be considered with caution and if possible double checked using a deterministic method.

## Introduction

Given a space *S* ⊆ ℝ^*n*^, we are interested in computing a set of *sample points* *s*_1_, …, *s*_*k*_ ∈ *S* that represent the space *S* and its properties. Thus, by randomly generating sample points this offers one approach to overcome the curse of dimensionality. For example, if *S* is a polyhedron, we can compute nearly uniformly distributed sampling points of *S* in polynomial time [1] and from this approximate the volume of the polyhedron [2, 3]. In contrast no deterministic polynomial-time algorithm can compute the volume of convex sets with less than exponential relative error in *n* [4, 5].

Thus, *sampling methods* are nowadays used in many application areas, for example in the analysis of flux spaces in genome-scale metabolic networks. A genome-scale metabolic network models the chemical reactions possible in an organism. Using the assumption that no internal substance can be over- or under-produced a flow problem is obtained. This then leads to a polyhedron of feasible flows (also called fluxes) through the network (called flux space):

$$F\;≔\;\{v\in {\mathbb{R}}^{n}:\;Sv=0,\ell \leq v\leq u\}$$

Here, *S* ∈ ℝ^*m*×*n*^ is called the stoichiometric matrix. It encodes for each reaction r ∈ R = {1,...,n} which and how much of the metabolites M = {1,...,m} are consumed resp. produced. ℓ, *u* are bounds on the reaction rates. Due to the size of these networks, deterministic methods to enumerate extreme points and thus a representative set of feasible flows [6, 7] are unpractical.

Therefore, tools and methods have been developed to sample the whole flux space [8–11] or only the extreme points [12] and then used to derive biological insights [13–16]. Typical properties that are analyzed are correlations between fluxes through different reactions [17–20], or the distribution of flux rates through a given reaction [21]

While points in polyhedra can be sampled efficiently (in theory), often additional constraints are added to make the results more biological reasonable. However, this often makes the space of feasible solutions non-convex and associated decision problems **NP**-hard. This is for example the case with “looplaw”-thermodynamic constraints [21, 22], here sloppily represented using the phrase “*v* thermo. feasible”:

T≔{v∈F:vthermo.feasible}

To test how well sampling works, we consider the scenario that we want to decide if the flux space has a given property. In Practical Obstructions to Sampling we consider the problem of determining if positive resp. negative flux through a reaction is possible. There, we show that with *artificial centering hit and run* (ACHR) [8, 23] we cannot determine this property correctly for several reactions in genome-scale metabolic networks. In particular, the artifacts are not only observed in non-convex flux spaces, but also for polyhedral flux spaces.

In Theoretical Obstructions to Sampling we show that for the non-convex flux space *T* this problem is not specific to ACHR, but more fundamental. Therefore, we generalize the problem to decide if a space *S* has a given property (formulated as a decision problem Prob) by using a sampling algorithm. We define the concept of *non-trivial polynomial time sampling algorithm* and show how it can be used to solve decision problems in randomized polynomial time. We show that if the decision property is NP-hard, then there exists no polynomial time sampling algorithm that samples *S* in a non-trivial way w.r.t. to the property formulated by Prob unless **NP** = **RP**, where **RP** is the class of problems that can be solved in randomized polynomial time [24].

## Practical Obstructions to Sampling

*Thermodynamic constraints* are an additional source of constraints that have been used in the analysis of metabolic networks [25–34] and were also used in sampling methods [21, 35, 36]. However, most thermodynamic constraints are computationally difficult, since they are non-convex. Here we use so-called “looplaw”-thermodynamic constraints.

**Fig 1:**
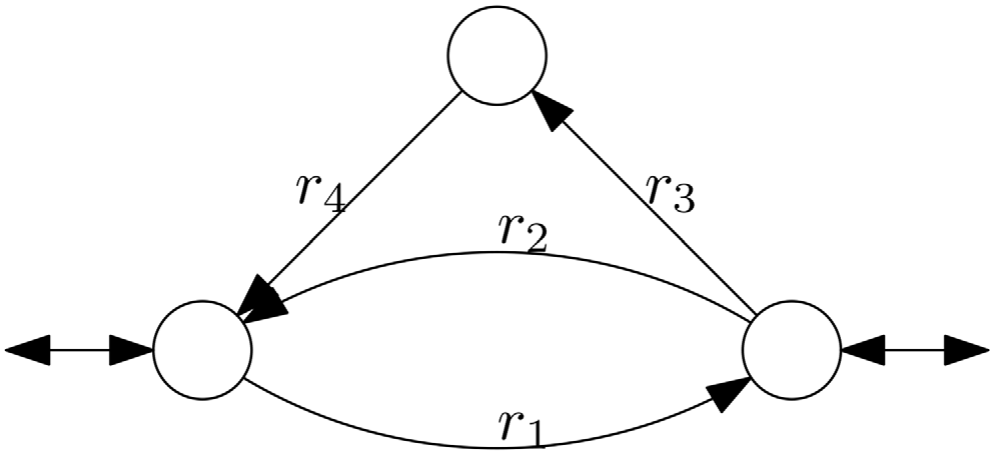
Toy network. Internal reactions *r*_1_, *r*_2_, *r*_3_, *r*_4_ are irreversible. By thermodynamics, it is not possible to have non-zero flux through *r*_1_ and also to have a non-zero flux through one of *r*_2_, *r*_3_ or *r*_4_ at the same time.

**Fig 2:**
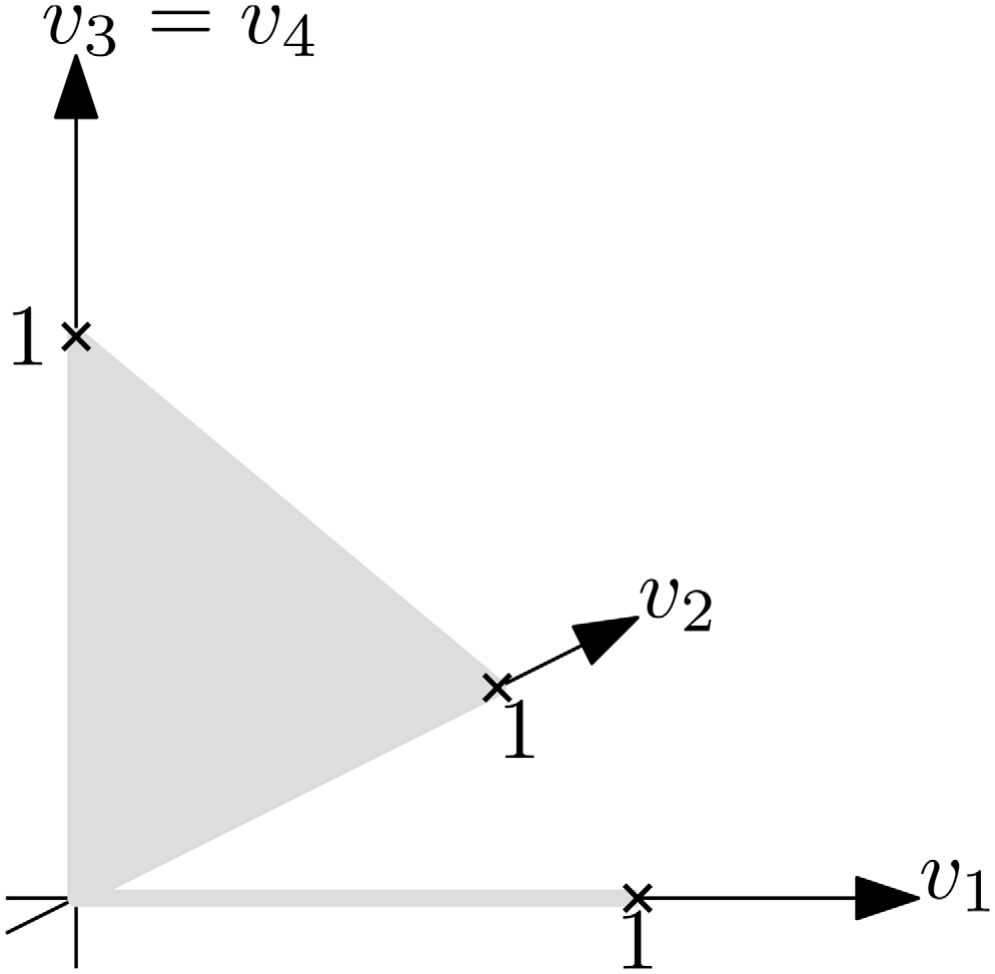
Flux space of toy network. The gray area denotes the flux space. In this example it was assumed that input/output flux values are constrained to at most 1. It can be seen that flux *v*_1_ through *r*_1_ is exclusive to fluxes *v*_2_ and *v*_3_ through *r*_2_ and *r*_3_ respectively. Since fluxes through *r*_2_ can be combined with fluxes through *r*_3_, the flux space with *v*_2_, *v*_3_ > 0 is two-dimensional, while the flux space with *v*_1_ > 0 is only one-dimensional. Hence, a uniform sample of the flux space would almost surely have zero flux through *r*_1_.

For the toy network shown in Fig 1 “looplaw”-thermodynamic constraints imply that positive flux through *r*_1_ and *r*_2_ at the same time is not possible, because they form a stoichiometrically balanced internal cycle. Similarly, positive flux through *r*_1_, *r*_3_, and *r*_4_ at the same time is also not possible. This implies that the flux space looks as shown in Fig 2. Clearly, this flux space is not convex. Furthermore, we observe that if we sample the flux space uniformly, we would not sample any flux with positive flux through *r*_1_, since the flux space with positive flux through *r*_1_ is 1-dimensional while the rest of the flux space is 2-dimensional.

For “looplaw”-thermodynamic constraints we showed that deciding if a reaction can carry positive flux is **NP**-hard [37]. Thus, according to our theoretical results in Theoretical Obstructions to Sampling, it should be harder to sample flux spaces with thermodynamic constraints than without. Therefore, we test how well we can predict the reactions with variable flux rates by sampling fluxes through the network. Finding all reactions with variable flux rate is called *flux variability analysis* (FVA). If it is applied without thermodynamic constraints, it can be solved efficiently using linear programming [38, 39]. With thermodynamic constraints, we can typically solve it in practice using mixed integer linear programming techniques (MILP) [21, 37]. For a good sampling algorithm we expect that for every reaction that can have positive flux (as determined by FVA) we also get samples whith positive flux through the reaction. Therefore, we count for how many of the reactions we fail this goal, i.e. where we do not even obtain a single sample with a positive flux through the reaction.

Of particular interest are the reactions $R^{C}$ of reactions contained in internal cycles, because they are affected by thermodynamic constraints. The reader is referred to [37] for more details and a precise definition. The reactions not contained in internal cycles $R^{NC}$ on the other hand should not be affected by “looplaw”-thermodynamic constraints [37]

### Method

To verify the impact of the theoretical results, we implemented the following computational experiment to analyze the difference between sampling with thermodynamic constraints and without thermodynamic constraints. For a given metabolic network with flux space *P* (with or without thermodynamic constraints) we do the following:

1. Sample *n* points in the flux space *P*.
2. Run flux variability analysis (FVA) on *P* and define:
  - $R_{+}$ ≔ reactions that can have positive flux,
  - $R_{-}$ ≔ reactions that can have negative flux.
3. From this we define the following 4 reaction classes:
  - $R_{+}^{C}≔R^{C}\cap R_{+}$
  - $R_{+}^{NC}≔R^{NC}\cap R_{+}$
  - $R_{-}^{C}≔R^{C}\cap R_{-}$
  - $R_{-}^{NC}≔R^{NC}\cap R_{-}$
4. For each reaction class $A\;\subseteq \;R_{+}$, we count the number of reactions for which we never sampled positive flux $n_{A}^{P}$ and then compute the ratio $r_{A}^{P}\;≔\;\frac{n_{A}^{P}}{|A|}$.
5. For each reaction class $A\;\subseteq \;R_{-}$, we count the number of reactions for which we never sampled negative flux $n_{A}^{P}$ and then compute the ratio $r_{A}^{P}\;≔\;\frac{n_{A}^{P}}{|A|}$.

We do this for the steady-state flux space *F* (without thermodynamic constraints) and for the thermodynamically constrained flux space *T*. Since positive lower bounds and negative upper bounds for reactions in internal cycles make it already **NP**-hard to find a thermodynamically feasible flux distribution, we set all positive lower bounds and all negative upper bounds to 0.

For the sampling method we chose to use the ACHR method implemented in the COBRA toolbox [40], since it is one of the most established tools for sampling flux spaces. They also offer a flag to activate thermodynamic constraints. Unfortunately this flag has no effect in the current version (2.0.5). Hence, we implemented a simple post-processing step to turn thermodynamically infeasible fluxes into thermodynamically feasible fluxes by deleting internal cycles [37]. To check that our results are not an artifact of our post-processing step, we also implemented the post-processing method suggested by Schellenberger et al. [21], where for each sample point a thermodynamically feasible flux vector is computed that minimizes the *L* ^1^-norm distance to the sample point. We remark that this method solves an MILP in the post-processing step and hence, cannot be considered a polynomial-time sampling method.

The sampling method was run with default parameters, except that the number of points per file is half the number of output points. This means that for each sample point ACHR performed at least 200 steps and potentially biased samples from the beginning were dropped. We choose 10000 output points, since this allowed to run the analysis in a couple of hours. In contrast, the variability of reactions (with and without thermodynamic constraints) can be computed deterministically using the FVA-method in [37] in a few minutes.

We selected a set of genome-scale metabolic networks based from the BiGG-database [41] as a test-set, since these networks are well curated and well established test-networks. We did not select *Human Recon 1*., since we were not able to run thermodynamically constrained flux variability analysis on it. Instead, we also added the more recent *E. coli* iJO1366 network [42]. Also, we did not select the *M. barkeri* network because the sampling algorithm from the COBRA toolbox crashed. The matlab scripts for the computations can be found in S1 code.

### Results

**Fig 3:**
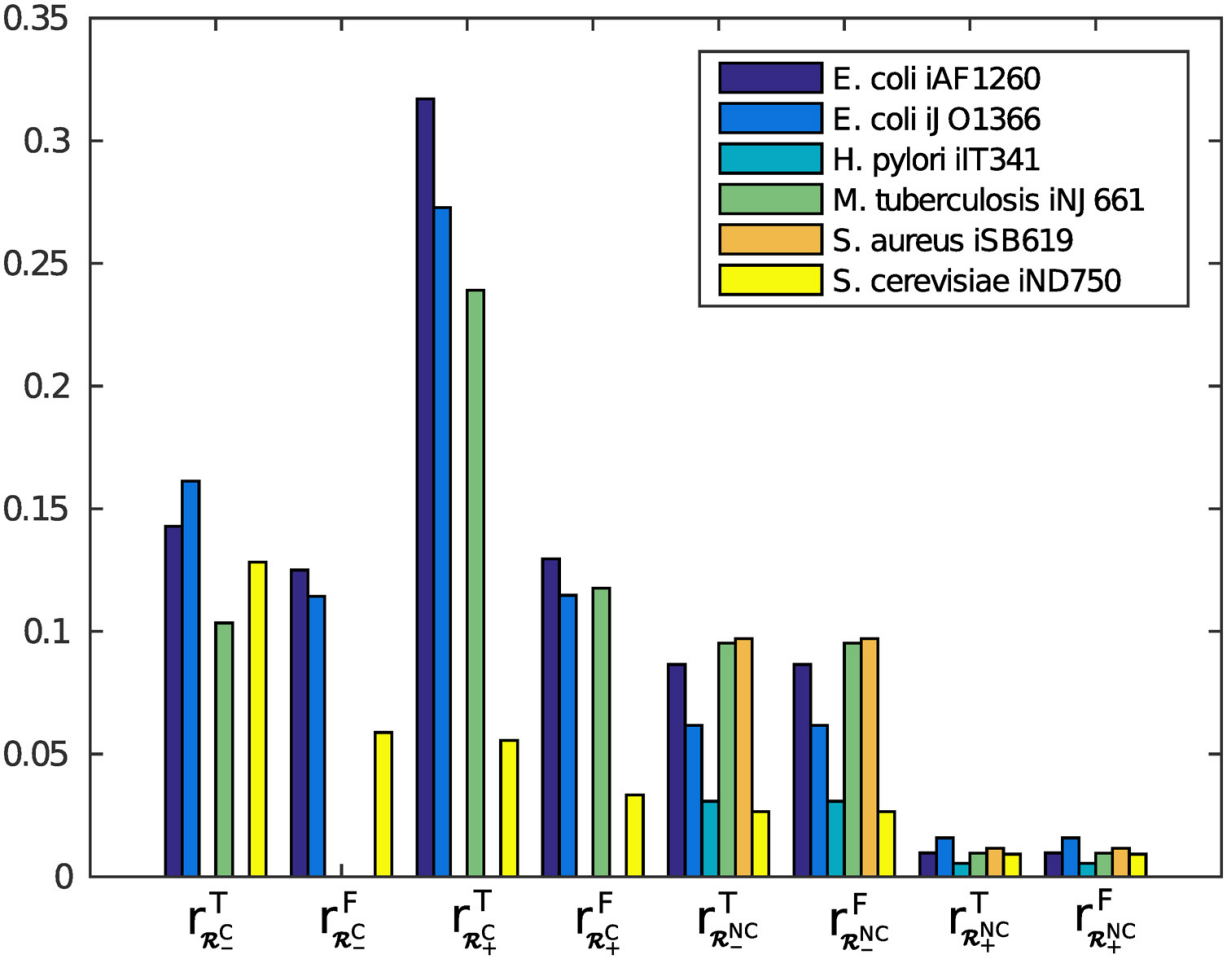
Sampling results with 10000 sample points. The *y*-axis shows the ratio for how many reactions that can have positive/negative flux the sampling method did not sample at least one such flux vector.

The computed results for 10000 samples using the cycle deletion method [37] can be seen in Fig 3. The *L* ^1^-minimization method [21] was not practically applicable for most of the test networks, because it took more than 10 minutes to compute the closest thermodynamically feasible flux vector for even the first sample point. Only for *H. pylori* iIT341 and *S. cerevisiae* iND750 this was a feasible approach. For these networks, however, already the simple cycle deletion method already managed to sample non-zero fluxes for all reactions in internal cycles that can have non-zero flux. Hence, these results are not separately shown.

We observe that the ratio of reactions where no positive/negative fluxes were sampled is larger for the case with thermodynamic constraints than without. Also, as expected, the results for $R^{NC}$ are the same for *F* and *T*.

However, we are surprised to find that even without thermodynamic constraints, we miss about 5% of all possible reaction directions. This shows us that ACHR might not be a very good approach for sampling steady-state flux spaces of genome-scale metabolic networks, as has also been observed in [43].

**Fig 4:**
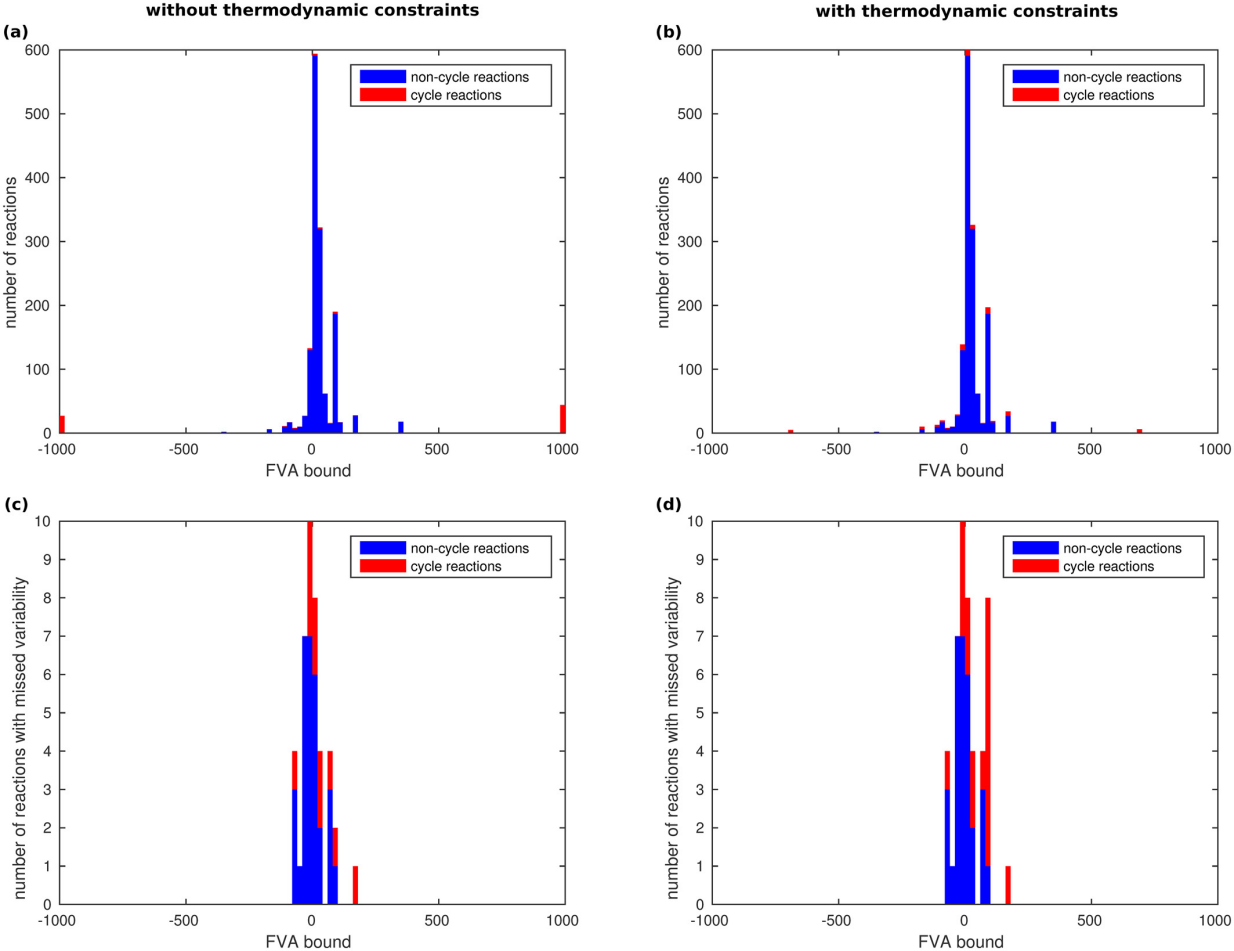
Distribution of *E. coli* iAF1260 flux variability that is missed by sampling. Histograms (a) and (c) are for the flux space without thermodynamic constraints, histograms (b) and (d) are with thermodynamic constraints. The bins in histograms (a) and (b) count the number of reactions with the respective lower and upper bounds computed by FVA. Bounds equal to 0 are not counted. Histograms (c) and (d) show, for the lower and upper bounds shown in (a) and (b), the number of reactions for which no negative resp. positive flux has been sampled. For all histograms, bin sizes of length 20 were chosen. We remark that zero flux is always possible. Therefore, the flux bounds directly relate to the possible flux range.

One potential cause for the bad performance of ACHR is that the flux space of metabolic networks is badly conditioned, i.e., that there exist reactions with very low variability and reactions with high variability. While this is indeed the case (see Fig 4a and 4b), it does not appear to be the primary cause, since not only reactions with very low variability are missed (see Fig 4c and 4d). From Fig 4 we also see that there exists an assymmetry between lower flux bounds and upper flux bounds, which could be a reason for the different results for positive and negative directions in Fig 3.

## Theoretical Obstructions to Sampling

Let Prob:J → {0, 1} be an **NP**-hard decision problem on a set J of inputs (commonly we use the set of words over the alphabet {0, 1} as input, i.e., J = {0, 1}* and the length of an input is just the length of the word). To solve Prob by sampling, we require that the structure of the sampling space represents Prob in a certain way. This we encode using a function X that maps every input I ∈ J into a subset of ℝ^*n*^, i.e. an element of the powerset of ℝ^*n*^ ($P({\mathbb{R}}^{n})$). This space X(I) will be the space from which we will draw samples. Note that we will make no assumptions on the size of *n* compared to the input size ∣*I*∣. Additionally, we use a test-function *f* that tests whether a point of the sample-space x ∈ X(I) has a non-trivial property, i.e. *f*(*I*, *x*) > 0.

**Definition 1 (Sampling space)** Given a decision problem Prob:J → {0, 1}, we call (X, f) a *sampling space* for Prob if $X\;:\;J\;\to \;P({\mathbb{R}}^{n})$ and $f\;:\;J\;\times \;{\mathbb{R}}^{n}\;\to \;\mathbb{R}$ satisfy:

- *f*(*I*, *x*) is continuous in *x* for all *x* ∈ ℝ^*n*^.
- *f*(*I*, *x*) can be computed in time polynomial in the encoding length of *I* and *x*. For *x* ∈ ℝ without a finite encoding length, we assume that *f*(*I*, *x*) is well defined, but its computation does not terminate.
- It holds for all I ∈ J that
  $$\text{PROB}\left(I\right)=\{\begin{array}{cc} 1 & \exists x\in X(I)\;:\;f(I,x)>0\\ 0 & \text{otherwise}. \end{array}$$

For example, let us assume that we want to know whether a given reaction r ∈ R can have positive flux in the flux space *F* of a given metabolic network. Thus, a problem instance *I* encodes a metabolic network N(I) and a target reaction *r*(*I*). It follows that (X, f), with X(I) := F(I) and *f*(*I*, *x*) ≔ pr_*r*(*I*)_(*x*) for all I ∈ J is a sampling space, where *F*(*I*) is the flux space of the metabolic network N(I) and pr_*r*_(*x*) denotes the flux through reaction *r* in the flux vector *x*.

Let (Ω, F, P) be a probability space, i.e., a sample space Ω, events F, and probability function *P*. It will serve us as the space from which we draw the seeds for the sampling algorithm. Here, we assume that the sampling method is given as a function $S\;:\;J\;\times \;\mathbb{N}\;\times \;\Omega \;\to \;{\mathbb{R}}^{n}$, i.e., for every time point we get a sample. With this formalism we want to capture the behavior of random-walk sampling methods that do a random walk through X(I) and can be run for arbitrarily long times to improve the sampling result. Classical sampling algorithms can also be captured by this formalism by iteratively running the sampling method and computing a consensus value. If the sampling algorithm did not produce a result for an (early) time point, it could simply return a default value. Since we will only consider asymptotic behavior, this will not be of any importance.

**Definition 2 (Feasible Sampling Algorithm)** $S\;:\;J\;\times \;\mathbb{N}\;\times \;\Omega \;\to \;{\mathbb{R}}^{n}$ is a *feasible sampling algorithm*, if there exists a polynomial *p* : ℕ → ℝ such that

S(I,k,ω)∈X(I)∀k≥p(|I|),I∈J,ω∈Ω

**Definition 3 (Polynomial Time Sampling Algorithm)** $S\;:\;J\;\times \;\mathbb{N}\;\times \;\Omega \;\to \;{\mathbb{R}}^{n}$ is a *polynomial time sampling algorithm* if there exists a polynomial *q* : ℕ × ℝ^+^ → ℝ^+^ and for every I ∈ J a random variable *X* : Ω → ℝ^*n*^ such that

- S(I, k, ω) for I ∈ J and *ω* ∈ Ω can be computed in time *O*(*k*),
- S(I, k, ⋅) → X in distribution for *k* → ∞, and
- S(I, k, ⋅)(*I*, *k*, ⋅) converges to *X* in polynomial time, i.e., for every closed set *A* ⊆ ℝ^*n*^ holds
  |P(S(I,k,·)∈A)-P(X∈A)|<ε
  for all *k* > *q*(∣*I*∣, *ɛ* ^−1^).

Assume there exists such a sampling method $S\;:\;J\;\times \;\mathbb{N}\;\to \;{\mathbb{R}}^{n}$ that samples the feasibility space X(I) of the **NP**-hard optimization problem Prob for each given instance I ∈ J in a non-trivial way, i.e., without losing any features (represented by *f*):

**Definition 4 (Non-trivial Sampling Algorithm)** $S\;:\;J\;\times \;\mathbb{N}\;\times \;\Omega \;\to \;{\mathbb{R}}^{n}$ is a *non-trivial sampling algorithm* w.r.t. $f\;:\;J\;\times \;{\mathbb{R}}^{n}\;\to \;\mathbb{R}$ if for every I ∈ J there exists a random variable *X* : Ω → ℝ^*n*^ such that

- S(I, k, ⋅) → X in distribution for *k* → ∞.
- If ∃x ∈ X(I) with *f*(*I*, *x*) > 0, then
  P(f(I,X)≤0)=t<1
  with $\frac{1}{1-t}\leq p(|I|)$ for a polynomial *p*.

We can then use S to construct a probabilistic algorithm that will decide Prob. The probabilistic algorithm that we are going to construct will belong to the class **RP** (randomized polynomial time) [24].

**Definition 5 (Complexity Class RP)** A decision problem *p* is in **RP** if there exists a probabilistic algorithm that

- runs in polynomial time,
- if the answer to *p* is NO, it outputs NO, and
- if the answer to *p* is YES, it outputs YES with probability at least $\frac{1}{2}$.

Since **RP** = **NP** is an open problem in theoretical computer science, it is very unlikely that a given probabilistic polynomial-time sampling algorithm of the thermodynamically constrained flux space actually solves the **RP** = **NP** problem. Hence, it is much more likely that the sampling algorithm samples the feasible flux space incompletely.

**Theorem 1** *Let* Prob: J → {0, 1} *be an* **NP**-*hard decision problem with sampling space* (X, f). *Unless* **RP** = **NP**, *there exists no feasible, non-trivial, polynomial time sampling algorithm* $S\;:\;J\;\times \;\mathbb{N}\;\times \;\Omega \;\to \;{\mathbb{R}}^{n}$.

Proof Assume there exists such a sampling algorithm. We construct an algorithm in **RP** for Prob.

For I ∈ J define *t*(*I*) ≔ *P*(*X* ∈ *A*(*I*)) for *A*(*I*) ≔ {*x* ∈ ℝ^*n*^ : *f*(*I*, *x*) ≤ 0}. Since *f*(*I*, ⋅) is continuous, it follows that *A*(*I*) is closed and Borel-measurable. Hence, *t*(*I*) is well defined.

By Def. 2 and Def. 3 there exist polynomials *k*_0_:ℕ → ℝ^+^, *q* : ℕ × ℝ^+^ → ℝ^+^ that satisfy for all I ∈ J, *ω* ∈ Ω, *k* ≥ *k*_0_(∣*I*∣),

S(I,k,ω)∈X(I) (1)

and for all *ɛ* > 0, *k* > *q*(∣*I*∣, *ɛ* ^−1^) (since *A* is closed)

P(S(I,k,·)∈A(I))-P(X∈A(I))<ε. (2)

We assume w.l.o.g. that *q*(*m*, *ɛ*) ≥ *k*_0_(*m*) for all *m* ∈ ℕ, *ɛ* ∈ ℝ^+^.

**Algorithm 1** Probabilistic Algorithm for Prob. *k*_0_ is the polynomial from Def. 2 and *q* is the polynomial from Def. 3.

$k=\text{max}\;\left\{q\;\left(|I|,\frac{2}{1-t}\right),k_{0}(I)\right\}$

choose random *ω* ∈ Ω

compute a sample $X_{K}:=S(I,\;k,\;\omega )$

**if** *f*(*I*, *X*_*k*_) ≤ 0 **then**

 return NO

**else**

 return YES

**end if**

**Lemma 1** *For a given input* I ∈ J *and* *t* ≥ *t*(*I*) *Algorithm 1 returns NO with probability at most* $\frac{t+1}{2}$ *if* Prob(*I*) = 1 *and it always returns NO if* Prob(*I*) = 0.

Proof **Case: There exists a** x ∈ X(I) **with** *f*(*x*) > 0:
  By (Eq 2) it follows for all *k* > *q*(∣*I*∣, *ɛ* ^−1^) that:
    P(f(S(I,k,·))≤0)<t(I)+ε≤t+ε
  By choosing $\varepsilon =\frac{1-t}{2}$, we obtain
    $$P\;\left(S\left(I,k,\cdot \right)\in A\right)\;<\;\frac{t+1}{2}.$$
    Thus, Alg. 1 will return NO although the correct answer is YES with probability at most $\frac{t+1}{2}$.
**Case** *f*(*x*) ≤ 0 **for all** x ∈ X(I):
  It follows that f(S(I, k, ω)) ≤ 0 for all *ω* ∈ Ω, *k* ≥ *k*_0_(*I*) by Def. 2. Hence, the answer of the algorithm will always be NO, if the correct answer is NO.

To prove that the problem would be in **RP**, we still have to increase the probability of YES in the positive case. This can be done by re-running the algorithm.

By Def. 4 there exists a polynomial *p* with $\frac{1}{1-t(I)}\leq p(|I|)$. We choose $t\;≔\;\frac{p(|I|)-1}{p(|I|)}$ and it follows that $\frac{1}{1-t}=p(|I|)$ and *t*(*I*) ≤ *t*. Hence, we can apply Lemma 1 without having to know *t*(*I*).

By construction of Alg. 1 the computation of *X*_*k*_ takes time $O(q(|I|,\frac{2}{1-t}))$. We observe that the encoding for the computed sample *X*_*k*_ is bounded by the computation time $O(q(|I|,\frac{2}{1-t}))$. Hence, by Def. 1 there exists a polynomial *g* such that the runtime of Alg. 1 is bounded by $O\;\left(g\;\left(|I|,q(|I|,\frac{2}{1-t})\right)\right)$.

To obtain a correct result if the correct answer is YES with probability at least $\frac{1}{2}$, we re-run the algorithm at least $\frac{1}{{\text{log}}_{2}\left(\frac{2}{t+1}\right)}$ times with independent choice of *ω* ∈ Ω for each run and return YES if one of the runs returned yes.

Since the probability of NO in one run is at most $\frac{t+1}{2}$, it follows that the probability for NO in all runs is at most

$${\left(\frac{t+1}{2}\right)}^{\frac{1}{{\text{log}}_{2}\left(\frac{2}{t+1}\right)}}=2^{\frac{{\text{log}}_{2}\left(\frac{t+1}{2}\right)}{{\text{log}}_{2}\left(\frac{2}{t+1}\right)}}=2^{-\frac{{\text{log}}_{2}\left(\frac{2}{t+1}\right)}{{\text{log}}_{2}\left(\frac{2}{t+1}\right)}}=\frac{1}{2}.$$

We can estimate the number of iterations by observing that

$$\begin{array}{cc} t & =\;\frac{p(|I|)-1}{p(|I|)}\\ \Rightarrow \frac{2}{t+1} & =\;\frac{2}{\frac{p(|I|)-1}{p(|I|)}+1}=\frac{2p(|I|)}{2p(|I|)-1}\\ \Rightarrow \frac{1}{\log_{2}(\frac{2}{t+1})} & =\;\frac{1}{\log_{2}(\frac{2p(|I|)}{2p(|I|)-1})}. \end{array}$$

Using the Theorem of l’Hopital we have

$$\begin{array}{cc} \lim_{p\to \infty }\frac{p^{-1}}{\ln(\frac{p}{p-1})} & =\lim_{p\to \infty }\frac{p^{-1}}{\ln p-\ln(p-1)}\\ & =\lim_{p\to \infty }\frac{-p^{-2}}{\frac{1}{p}-\frac{1}{p-1}}\\ & =\lim_{p\to \infty }\frac{-p^{-2}}{\frac{p-1-p}{p(p-1)}}\\ & =\lim_{p\to \infty }p^{-2}\left(p^{2}-p\right)\\ & =1 \end{array}$$

Hence, we can bound the number of iterations by

$$\frac{1}{\log_{2}(\frac{2}{t+1})}=\frac{1}{\log_{2}(\frac{2p(|I|)}{2p(|I|)-1})}=O(p(|I|)).$$

Thus, we get a YES if the correct answer is YES with probability at least $\frac{1}{2}$ after a running time of

$$\begin{array}{cc} & O\;(g\;(|I|,q\;(|I|,\frac{2}{1-t})))\frac{1}{\log_{2}(\frac{2}{t+1})}\\ \leq & O\;(g\;(|I|,q\;(|I|,2p(|I|)))p\left(|I|\right)). \end{array}$$

We have shown under the assumption of the existence of a sampling algorithm with the given properties that Prob is in **RP**. Since Prob is also **NP**-hard, the existence of such a sampling algorithm implies **RP** = **NP**. Hence, no such sampling algorithm can exist if **RP** ≠ **NP**.

## Discussion

We observe that the conditions that we require for Thm. 1 on the sampling algorithm are very weak. We do not require uniform distribution, we only require that with some polynomially small probability we also sample fluxes unequal to zero in our target distribution and that we converge in polynomial time to this target distribution.

Assuming **RP** ≠ **NP**, it follows that for every sampling algorithm on the thermodynamic flux space there exist networks where the algorithm has one of the following properties:

- The sampling algorithm does not converge in polynomial time to the target distribution, or
- the target distribution is trivial (i.e., the probability of sampling 0 is 1).

Of course, we may be lucky and the algorithm actually samples a non-trivial distribution for the input networks. However the result says that there are networks for which the sampling algorithm will only sample 0 fluxes for some reactions and indeed, we saw that this happens also in practice not only for sampling the thermodynamically constrained flux space but also the ordinary steady-state flux space. It might be a property of the ACHR method that even for the ordinary steady-state flux space only 0 fluxes for some reactions are sampled, because in theory classical hit and run sampling (with appropriate rounding to remove the heterogeneous scales of metabolic networks) is guaranteed to sample uniformly [1]. De Martino et al. [43] showed that indeed ACHR seems to have problems with high-dimensional instances, like 500 dimensional uniform hypercubes. Other sampling methods, e.g. loopy-belief propagation [10, 11] or poling-based methods [9] might not have these problems.

Unreliable sampling is very critical, since we then may be led to the false assumption that the reaction is never used, although it actually could be. To make sure that such results are true, it is essential to verify them with a deterministic method. In the case of deciding whether flux is possible through a given reaction, we can decide this by solving an optimization problem [21, 37].

We have shown that sampling artifacts happen for the flux variability problem with thermodynamic constraints (and in practice they even happen without thermodynamic constraints with ACHR sampling). However, sampling is used to check a wide variety of different properties. Although the result does not directly imply that sampling results for these other properties are unreliable as well, caution is highly advised. For example, consider correlation / flux coupling analysis [20]. If a reaction always carries zero flux in all samples by an artifact, although it can also carry non-zero flux, it follows that this reaction seems uncorrelated to all other reactions. However, it may very well be correlated / coupled. In Fig 1, we see such an example. Assume the flux space (see Fig 2) is sampled using a uniform distribution. Then, we will almost surely never sample non-zero flux through reaction *r*_1_. Correlation analysis would yield that flux through *r*_1_ is uncorrelated (they are even independent) to flux through *r*_2_ and *r*_3_, although the fluxes are actually exclusive (e.g. *r*_1_ and *r*_2_ cannot carry flux at the same time). On the other hand, if reaction *r*_2_ would be removed (because it is simply the aggregation of *r*_3_ and *r*_4_) the result would change significantly. Then, with uniform sampling, positive fluxes through *r*_1_ and through *r*_3_ would be sampled with equal probability.

## Conclusion

Although sampling has been used successfully for the analysis of many different kind of problems, the results obtained by sampling should be used with caution. In the field of metabolic network analysis in particular, we showed (assuming **RP** ≠ **NP**) that for every polynomial-time sampling method obeying thermodynamic constraints there exist networks for which the sampling method will produce artifacts. Hence, qualitative results obtained by sampling complex spaces should always be double checked by a different method.

## Supporting Information

S1 Code Source code. Matlab scripts used to compute the results in the manuscript.
(ZIP)
